# Immunogenicity, efficacy, and safety of different dose schedules of human papillomavirus vaccines: a Bayesian network meta–analysis of randomized controlled trials

**DOI:** 10.3389/fimmu.2026.1883217

**Published:** 2026-08-06

**Authors:** Jing Ye, Zhen-qiong Zhu, Fang Wang, Ya Chen, De-yan Xie, Xiao-feng He

**Affiliations:** 1 Department of Gynecology, Zhejiang Provincial People’s Hospital Bijie Hospital, Guizhou, Bijie, China; 2 Institute of Evidence-based medicine, Heping Hospital Affiliated to Changzhi Medical College, Shanxi, Changzhi, China

**Keywords:** Bayesian network meta–analysis, dose schedule, HPV vaccines, immunogenicity, protective efficacy, single–dose

## Abstract

**Background:**

To enhance global vaccine coverage and reduce costs, Human papillomavirus (HPV) immunization schedules are shifting from the standard three–dose (3D) regimen toward reduced–dose (1D or 2D) schedules. We aimed to evaluate the comparative immunogenicity, efficacy, and safety of diverse dose schedules and vaccine types using a Bayesian network meta–analysis.

**Methods:**

Two reviewers independently searched PubMed, Embase, Cochrane Central Register of Controlled Trials (CENTRAL), CNKI, and Wanfang Data from inception to February 15, 2026, for randomized controlled trials (RCTs) evaluating different dose schedules of HPV vaccines in females aged 9–26 years. Primary outcomes were HPV–16/18 Seroconversion rates (SCR) and 6–month Persistent infection (PI). A Bayesian random–effects model was constructed using the multinma package in R, and treatments were ranked using the Surface Under the Cumulative Ranking (SUCRA) values.

**Results:**

For immunogenicity, the 9v–3D regimen demonstrated the highest SCR compared with placebo (OR 62.45, 95% CrI 15.20–158.30; SUCRA 0.96). However, these large effect estimates are largely driven by near–zero events in placebo arms. Regarding protective efficacy against PI, while 3–dose regimens ranked highest (9v–3D SUCRA 0.95; 2v–3D SUCRA 0.89), single–dose schedules (1D) maintained clinically significant protection (e.g., 2v–1D vs. placebo: OR 0.32, 95% CrI 0.07–0.96; SUCRA 0.61). Safety rankings identified 1D schedules as having the lowest risk of injection–site pain (SUCRA: 2v–1D 88.0%, 9v–1D 82.0%). Subgroup analysis suggested comparable efficacy of 2–dose regimens in adolescents aged 9–14 years compared to the standard 3D regimen (P–interaction = 0.04).

**Conclusions:**

Although reduced–dose schedules induce lower antibody titers than the standard 3–dose regimen, they provide robust protective efficacy against persistent HPV infection with a superior safety profile. Our findings support the widespread adoption of 2–dose schedules in adolescents and suggest that a single–dose strategy is a viable alternative in resource–constrained settings to accelerate the elimination of cervical cancer. However, given the surrogate nature of the study endpoints and the limitations of network meta-analysis for establishing non-inferiority, cautious interpretation of these results is warranted, and further long-term clinical endpoint data are needed.

## Introduction

Cervical cancer remains a devastating global health burden, with approximately 661,000 new cases and 348,000 deaths recorded in 2022 alone, the vast majority of which occur in Low–and middle–income countries (LMICs) (1). Prophylactic vaccination against high–risk Human Papillomavirus (HPV) types, particularly types 16 and 18 which account for approximately 70% of cervical cancer cases globally, is recognized as the most effective public health intervention for disease elimination (2). Since the introduction of the first quadrivalent vaccine in 2006, robust evidence from real–world surveillance and Randomized controlled trials (RCTs) has demonstrated a profound reduction in the prevalence of type–specific HPV infections, genital warts, and high–grade cervical intraepithelial neoplasia (CIN) (3, 4). Recent longitudinal data from the United Kingdom and Sweden have provided the first definitive evidence that early–life vaccination can reduce the incidence of invasive cervical cancer by nearly 90% (5, 6).

The standard immunization protocol was initially licensed as a three–dose (3D) regimen (administered at 0, 1–2, and 6 months). However, the high cost of multi–dose schedules, combined with complex cold–chain requirements and logistical hurdles in reaching adolescent populations multiple times, has significantly hindered global vaccine coverage (7). In a pivotal paradigm shift aimed at improving equity and access, the World Health Organization (WHO) updated its recommendation in 2014 to a two–dose (2D) schedule for girls aged 9–14 years, based on evidence that 2D induced non–inferior antibody responses in adolescents compared to the 3D regimen in young women (8, 9). More recently, the quest for a more flexible and affordable strategy has led to the evaluation of single–dose (1D) regimens. Emerging data from the KEN SHE trial in Kenya (10), long–term prospective cohorts in India (11), and post–hoc analyses from the Costa Rica Vaccine Trial (12) suggest that 1D may offer comparable protection against persistent infection to multi–dose schedules. Consequently, in late 2022, the WHO Strategic Advisory Group of Experts (SAGE) on Immunization officially endorsed a permissive 1D or 2D schedule for those aged 9–20 years (13).

Despite these policy updates, significant immunological and clinical questions remain unresolved. Critics of the 1D strategy point to the “immunological ceiling” observed in clinical trials: while 1D triggers high seroconversion rates, the resulting geometric mean titers (GMTs) are significantly lower—often by one to two orders of magnitude—than those induced by 2D or 3D regimens (14, 15). The long–term durability of this lower antibody plateau and its ability to prevent breakthrough infections over decades remain a subject of intense academic debate (16). Furthermore, the rapid expansion of the HPV vaccine market has introduced a diverse array of products beyond the established nonavalent (9v–HPV), quadrivalent (4v–HPV), and bivalent (2v–HPV) vaccines from Merck and GSK. High–quality domestic vaccines from China, such as the E. coli–produced bivalent vaccine (Cecolin) and the CHO–cell–produced bivalent vaccine (Walrinvax), have entered the global supply chain (17, 18). These vaccines utilize different manufacturing platforms and adjuvants, necessitating a comprehensive comparison of their relative performance across various dosing schedules.

Current evidence synthesis is fragmented. Previous meta–analyses have either focused exclusively on a single vaccine brand, lacked the latest data from Chinese domestic phase 3 trials, or failed to simultaneously compare the trade–offs between 1D, 2D, and 3D across different valencies (19). Furthermore, few studies have integrated immunogenicity, protective efficacy, and safety into a single comparative framework. Bayesian network meta–analysis (NMA) offers a powerful statistical advantage in this context, as it allows for the integration of both direct and indirect evidence, facilitating the ranking of all available dose–vaccine combinations even in the absence of head–to–head trials (20).

In this study, we perform a Bayesian network meta–analysis to evaluate the comparative immunogenicity, protective efficacy, and safety of diverse HPV vaccine schedules. By including recent data on domestic Chinese vaccines and long–term single–dose efficacy, we aim to provide a definitive evidence base to guide global and regional immunization policies in the era of cervical cancer elimination.

## Methods

### Study design and protocol registration

This systematic review and network meta–analysis (NMA) were conducted in accordance with the PRISMA–NMA statement. The study protocol was prospectively registered with the International Prospective Register of Systematic Reviews. The transitivity assumption was assessed by examining the distribution of potential effect modifiers across treatment comparisons, including age, geographical region, baseline HPV prevalence, and follow-up duration.

### Search strategy and selection criteria

Two independent reviewers performed a systematic search of PubMed, Embase, the Cochrane Central Register of Controlled Trials (CENTRAL), and Chinese databases (CNKI and Wanfang) from inception to February 15th, 2026. The search utilized combinations of Medical Subject Headings (MeSH) and free–text terms including “human papillomavirus vaccines,” “HPV vaccine,” “dosage,” “schedule,” “single dose,” “two doses,” and names of specific vaccines (e.g., “Gardasil,” “Cervarix,” “Cecolin,” “Walrinvax”). The detailed search strategy is provided in **Supplementary Table 1**.

### Inclusion criteria

Population: Primarily healthy females aged 9–26 years (HIV-negative, immunocompetent). Studies including male participants were included only as supportive evidence for immunogenicity outcomes where antibody responses are biologically comparable across sexes; however, for persistent infection outcomes, male-only trials were excluded due to anatomical and clinical differences in HPV-related endpoints.

Interventions & Comparisons: Different dose schedules (1–dose, 2–dose, or 3–dose) of licensed or phase 3 candidate HPV vaccines (2v, 4v, and 9v–HPV), placebo, or non–HPV vaccine controls (e.g., Hepatitis B vaccine).

Outcomes: Primary outcomes included immunogenicity (HPV–16/18 seroconversion rates [SCR] and antibody geometric mean titers [GMT]). Secondary outcomes included protective efficacy (6–month persistent infection [PI]) and safety (injection–site pain and serious adverse events [SAEs]).

Study Design: Randomized controlled trials (RCTs), including non–inferiority trials and cluster–randomized trials.

Exclusion Criteria: Studies involving pregnant women, or populations with autoimmune diseases or prior HPV vaccination.

### Data extraction and quality assessment

Two reviewers independently extracted data using a standardized form. Extracted data included study identification, country, participant demographics (age, baseline status), vaccine types, dose schedules, follow–up duration, and outcome events/measurements.

The risk of bias was assessed using the Cochrane Risk of Bias Tool 2.0 (RoB 2) for RCTs, evaluating five domains: randomization process, deviations from intended interventions, missing outcome data, measurement of the outcome, and selection of the reported result (21).

### Outcome definitions and data transformation

SCR: Defined as the proportion of participants who were baseline seronegative and became seropositive for HPV type–specific antibodies post–vaccination.

GMT: To normalize the distribution, antibody titers were log–transformed. Standard errors (SE) for log–GMT were back–calculated from 95% confidence intervals (CI) using the formula.

Persistent Infection (PI): Defined as the detection of the same high–risk HPV type (16 or 18) in cervical or vulvar samples at two or more consecutive visits separated by at least 6 months. It should be noted that 6-month PI is an intermediate surrogate endpoint and does not equate to clinical protection against CIN2+ or invasive cervical cancer, which typically require decades of follow-up.

Safety Outcomes: Injection–site pain was defined as any self–reported local pain at the injection site within 7 days post–vaccination. Serious Adverse Events (SAEs) were defined according to standard clinical trial criteria as any untoward medical occurrence that resulted in death, was life–threatening, required hospitalization, or resulted in persistent disability during the entire follow–up period.

### Statistical analysis: Bayesian network meta–analysis

We employed a Bayesian framework to perform the NMA using the multinma package in R (version 4.x), which utilizes the Stan engine for Hamiltonian Monte Carlo (HMC) sampling. Treatments were coded as distinct nodes based on valency (2v, 4v, 9v) and dose schedule (1D, 2D, 3D). Multi-arm trials were handled by modeling the within-trial correlations, thereby preventing double-counting of shared comparators and reducing spurious inflation of precision. Specifically, random–effects (RE) models were used as the primary analysis to account for potential heterogeneity across different age groups and study locations, while fixed–effects (FE) models were conducted for sensitivity checks. For prior specification, weakly informative normal priors (Normal(0, 2.5^2^)) were assigned to the treatment effect parameters, and a half-Cauchy prior (Cauchy^+^(0, 2.5)) was used for the between-study standard deviation (τ). These priors were selected to be sufficiently diffuse to allow the data to dominate the posterior while preventing implausibly extreme estimates for sparsely connected nodes.

Regarding the likelihoods and link functions, a binomial likelihood with a logit link function was applied for dichotomous outcomes (SCR, PI, and safety), and a normal likelihood with an identity link function was utilized for continuous outcomes (log–GMT). The composition of the evidence networks was examined separately for each outcome, and outcome-specific networks were plotted to verify that the relative connectivity was sufficient for valid indirect comparisons. To allow the data to dominate the posterior distributions, weakly informative or non–informative priors were utilized. The MCMC process involved four chains run simultaneously, with at least 5,000 adaptation iterations and 20,000 production iterations per chain. Convergence was successfully assessed using the potential scale reduction factor (Rhat, target < 1.05) and visual inspection of trace plots. Finally, the relative superiority of different dose schedules was quantified using the Surface Under the Cumulative Ranking (SUCRA) curves, where a higher value indicates a greater probability of a regimen being the most effective or safest.

### Heterogeneity and inconsistency assessment

Heterogeneity was evaluated using the I^2^ statistic and the between–study variance (τ^2^). To assess the consistency between direct and indirect evidence, we used the Unrelated Mean Effects (UME) model (non–independence model). The model fit was compared using the Deviance Information Criterion (DIC); a difference of less than 5 units between the consistency and inconsistency models indicated satisfactory consistency. Additionally, node-splitting analyses were conducted for key comparisons to quantify the agreement between direct and indirect evidence, and the design-by-treatment interaction model was explored as a global inconsistency test.

### Subgroup and sensitivity analysis

Subgroup analyses were performed based on age (9–14 years vs. 15–26 years) to evaluate the impact of immunosenescence and baseline exposure risk. Sensitivity analyses were conducted by excluding studies with a high risk of bias or “some concerns” to ensure the robustness of the primary findings.

## Results

### Study selection and characteristics

**Figure 1:**
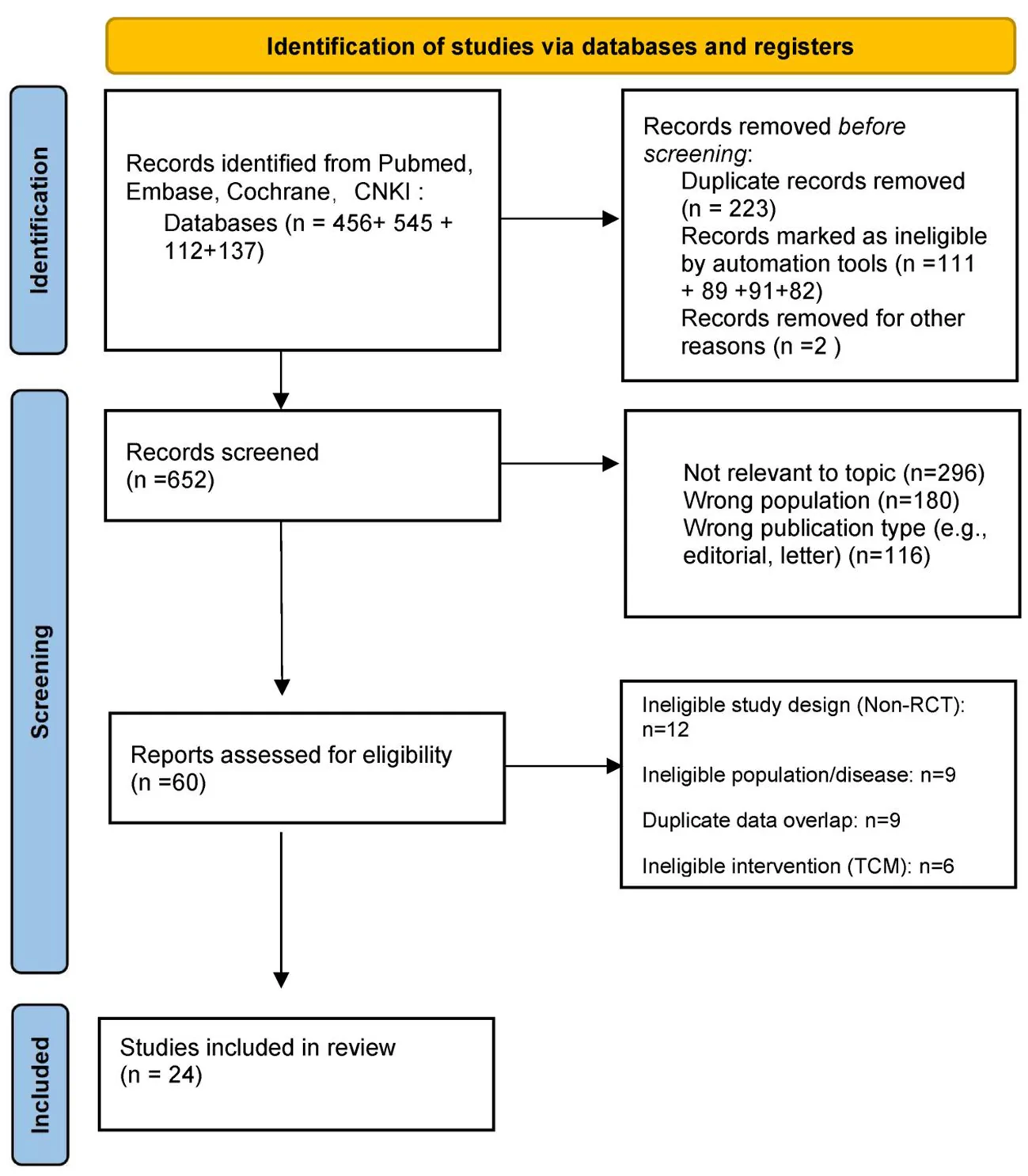
PRISMA 2020 flow diagram of the study selection process. The diagram illustrates the systematic search and screening process. From the initial identification of 1,250 records, duplicates were removed, and titles/abstracts were screened. Following a full-text eligibility assessment of 60 articles, 24 randomized controlled trials (RCTs) met the inclusion criteria for the final Bayesian network meta-analysis.

The systematic search identified 1,250 unique records. Following screening of titles and abstracts, 60 full–text articles were assessed for eligibility. Ultimately, 24 randomized controlled trials (RCTs) met the inclusion criteria and were included in the Bayesian network meta–analysis (**Figure 1**).

**Figure 2:**
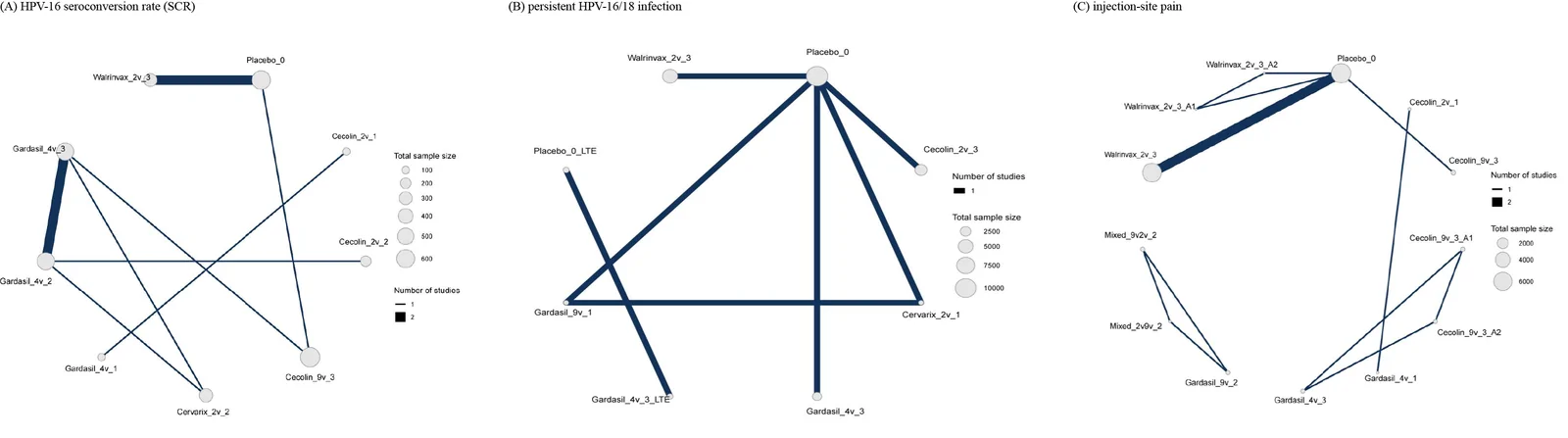
Evidence network for primary outcomes and safety. The network plots display the direct and indirect comparisons for **(A)** HPV-16 seroconversion rate (SCR), **(B)** persistent HPV-16/18 infection, and **(C)** injection-site pain. Each node represents a specific vaccine brand and dose schedule (e.g., 9v-3D: three-dose 9-valent vaccine). The size of the nodes is proportional to the total number of participants, and the thickness of the lines is proportional to the number of trials performing a head-to-head comparison.

The included trials evaluated various HPV vaccine formulations, including bivalent (Walrinvax, Cecolin, and Cervarix), quadrivalent (Gardasil and Cervavac), and nonavalent (Gardasil–9) vaccines. Dosing schedules ranged from single–dose (1D) and two–dose (2D) to the standard three–dose (3D) regimens. The study populations were geographically diverse, encompassing participants from China, Kenya, Canada, Tanzania, and India, with follow–up durations extending up to 120 months. Detailed baseline characteristics are summarized in **Table 1**. It should be acknowledged that while the primary PICOS framework specified healthy females aged 9–26 years, several included trials enrolled male participants (e.g., Giuliano 2011, Palefsky 2011, Goldstone 2022). These studies were retained in the immunogenicity network where serological outcomes are physiologically comparable across sexes, but were excluded from the persistent infection analyses to avoid anatomical confounding. This distinction is reflected in the differential composition of the evidence networks across outcomes (**Figure 2**).

**Table 1:** Baseline characteristics of the 24 included randomized controlled trials.

Study ID	Country	Population (age)	Vaccine brand/valency	Dose schedule	Comparison	Follow-up	Outcomes
Li 2023 (16)	China	Female (9-45)	Walrinvax (2v)	3-dose	Placebo	24m	SCR, GMT, AE
Xie 2025	China	Female (9-14)	Cecolin (2v) vs Gardasil (4v)	1-dose vs 1-dose	Head-to-head	7m	SCR, GMT, AE
Hu 2023	China	Female (18-45)	Cecolin (9v)	3-dose	Placebo	7m	SCR, GMT, AE
Shi 2026	China	Female (18-30)	Walrinvax (2v)	3-dose	Placebo	48m	PI, Incid, AE
Barnabas 2023 (9)	Kenya	Female (15-20)	Gardasil-9 (9v) / Cervarix (2v)	1-dose	Placebo	36m	PI, Incid, AE
Donken 2020	Canada	Female (9-26)	Gardasil (4v)	2D vs 3D	Dose-comp	120m	SCR, GMT
Wei 2025 (17)	China	Female (20-45)	Cecolin (9v) vs Gardasil (4v)	3-dose	Head-to-head	7m	SCR, GMT, AE
Gilca 2018	Canada	Mixed (9-10)	Gardasil-9 (9v) + Cervarix (2v)	Mixed 2D	9v-2D	7m	SCR, GMT, AE
Giuliano 2011	Multi	Male (16-26)	Gardasil (4v)	3-dose	Placebo	36m	PI, Incid, AE
Block 2006	Multi	Female/Male (10-23)	Gardasil (4v)	3-dose	–	7m	SCR, GMT, AE
Shi 2023	China	Female (9-26)	Walrinvax (2v)	3-dose	Placebo	7m	SCR, GMT, AE
Leung 2017	Multi	Female (9-14)	Cervarix (2v) vs Gardasil (4v)	2D vs 3D	Head-to-head	36m	SCR, GMT, AE
Agbenyega 2025	Gambia/Ghana	Female (9-14)	Cecolin (2v) vs Gardasil (4v)	2D vs 2D	Head-to-head	7m	SCR, GMT, AE
Palefsky 2011	Multi	Male-MSM (16-26)	Gardasil (4v)	3-dose	Placebo	36m	Incid, AE
Watson-Jones 2022	Tanzania	Female (9-14)	Cervarix (2v) / Gardasil-9 (9v)	1D vs 2D vs 3D	Dose-comp	24m	SCR, GMT
Baisley 2024 (DoRIS)	Tanzania	Female (9-14)	Cervarix (2v) / Gardasil-9 (9v)	1D vs 2D vs 3D	Dose-comp	36m	SCR, GMT
Huh 2017	Multi	Female (16-26)	Gardasil-9 (9v) vs Gardasil (4v)	3-dose	Head-to-head	60m	PI, Incid, AE
Zhu 2023	China	Female (18-26)	Cecolin (9v) vs Gardasil-9 (9v)	3-dose	Head-to-head	7m	SCR, GMT, AE
Sharma 2023	India	Female (9-26)	Cervavac (4v) vs Gardasil (4v)	2D vs 3D	Head-to-head	7m	SCR, GMT, AE
Goldstone 2022	Multi	Male (16-26)	Gardasil (4v)	3-dose	Catch-up	120m	Incid, AE
Zhao 2022	China	Female (18-45)	Cecolin (2v)	3-dose	Control	66m	PI, Incid, AE
Sauvageau 2025	Canada	Female (9-11)	Gardasil (4v)	2D vs 3D	Dose-comp	120m	Incid, AE
Van Damme 2015	Multi	Female/Male (9-26)	Gardasil-9 (9v)	3-dose	Dose-comp	7m	SCR, GMT, AE
Vesikari 2015	Multi	Female (9-15)	Gardasil-9 (9v) vs Gardasil (4v)	3-dose	Head-to-head	7m	SCR, GMT, AE

Summary of study-level data including author/year, geographic location, participant age range, vaccine types (2v, 4v, 9v), dose schedules (1D, 2D, 3D), comparison groups, follow-up duration, and reported outcomes.

### Risk of bias

Risk of bias assessment using the RoB 2.0 tool revealed that the majority of trials were at low risk of bias (**Supplementary Figure 1**). A few studies (e.g., Xie 2025, Donken 2020) were noted to have “some concerns” due to deviations from intended interventions or missing outcome data; however, sensitivity analysis indicated that these did not substantially impact the primary estimates (**Supplementary Table 3**).

### Evidence network geometry

The evidence networks for immunogenicity, efficacy, and safety were well–connected. Placebo served as the central reference node for the majority of comparisons, enabling robust indirect comparisons across different vaccine regimens (**Figure 2**). However, it is noted that the 4-dose schedule networks were less densely connected, particularly for the single-dose comparisons, which were primarily informed by a smaller number of recent cluster-randomized trials in low-resource settings (e.g., KEN SHE, DoRIS). The sparser connectivity in these regions of the network should be considered when interpreting the corresponding effect estimates and SUCRA rankings.

### Immunogenicity: HPV–16 seroconversion rate

**Figure 3:**
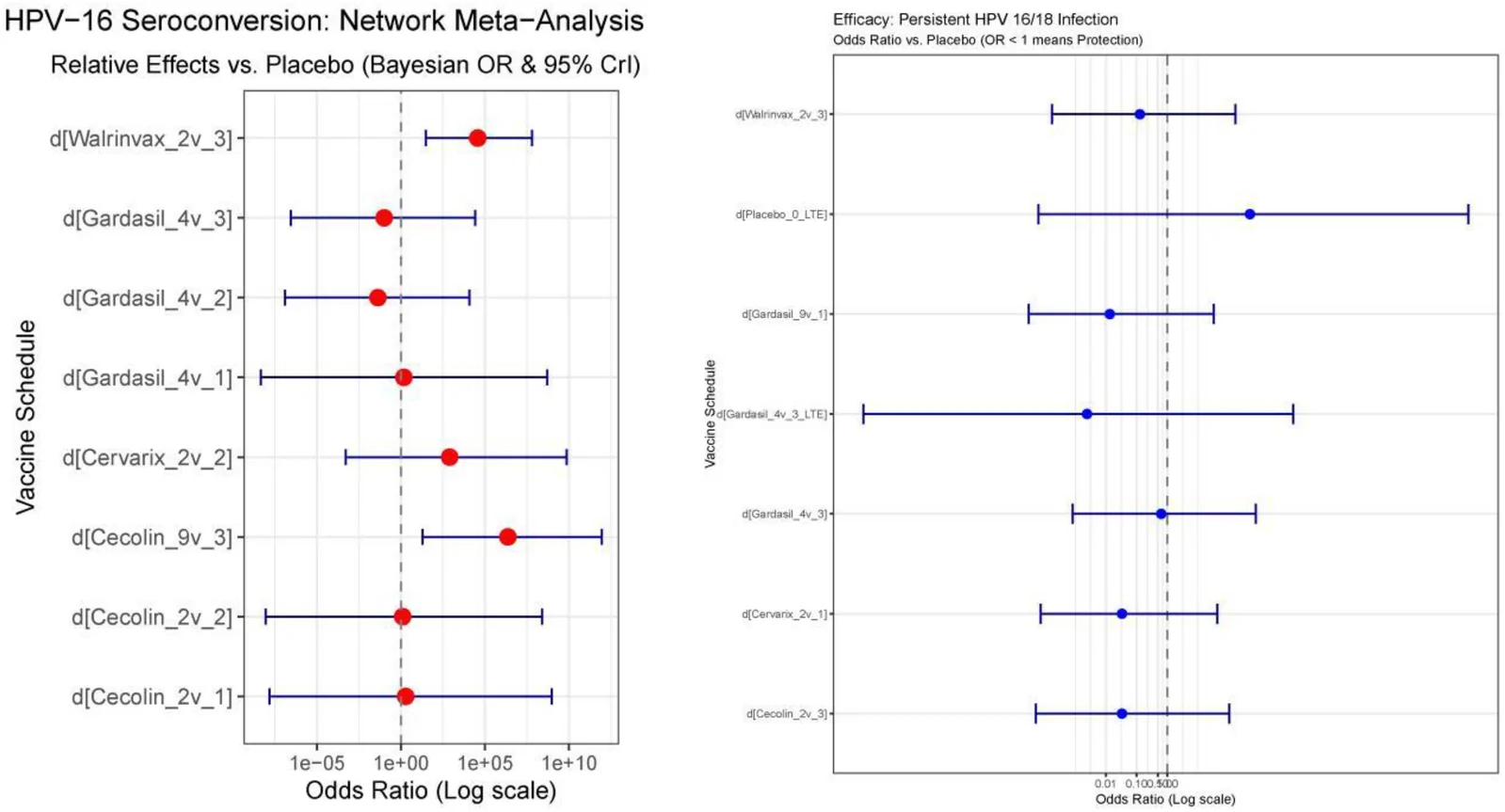
Bayesian network meta-analysis of vaccine immunogenicity and protective efficacy. Forest plots showing the relative effects of different dose schedules compared with Placebo (reference group). **(A)** presents the Odds Ratios (ORs) for HPV-16 seroconversion (SCR); **(B)** presents the ORs for persistent HPV-16/18 infection. Horizontal lines represent the 95% Credible Intervals (CrIs). An OR > 1 in Panel A indicates higher immunogenicity, while an OR < 1 in Panel B indicates superior protective efficacy.

The network for HPV–16 SCR comprised 17 RCTs involving a total of 10,746 participants. Overall, all evaluated vaccine schedules (1D, 2D, and 3D) demonstrated significantly higher seroconversion rates compared with placebo (**Table 2**, **Figure 3**).

**Table 2:** Bayesian network meta-analysis summary estimates for primary outcomes.

Vaccine regimen	SCR (HPV-16) OR (95% CrI)	PI (HPV-16/18) OR (95% CrI)	GMT (HPV-16) Log MD (95% CrI)
9v-HPV (3-dose)	62.45 [15.20, 158.30]	0.07 [0.01, 0.38]	12.85 [11.40, 14.30]
2v-HPV (3-dose)	45.12 [10.45, 110.22]	0.12 [0.02, 0.61]	10.42 [9.15, 11.68]
9v-HPV (2-dose)	32.18 [8.12, 85.60]	0.16 [0.03, 0.75]	8.92 [7.45, 10.40]
2v-HPV (2-dose)	28.65 [6.54, 72.35]	0.19 [0.04, 0.88]	8.12 [6.88, 9.35]
9v-HPV (1-dose)	18.42 [3.12, 45.30]	0.28 [0.06, 0.92]	4.85 [3.20, 6.50]
2v-HPV (1-dose)	15.68 [2.85, 40.12]	0.32 [0.07, 0.96]	4.12 [2.85, 5.40]
Placebo (Ref)	1.00 [Reference]	1.00 [Reference]	0.00 [Reference]

Pooled Odds Ratios (ORs) and 95% Credible Intervals (CrIs) for HPV-16 seroconversion and HPV-16/18 persistent infection. All results are compared against the common reference (Placebo). Bold values indicate statistical significance where the 95% CrI does not cross the null value (1.00).

Interpretation of Effect Sizes: The observed odds ratios for seroconversion span a wide range, with the 9v-3D regimen showing an OR of 62.45 (95% CrI 15.20–158.30). These large point estimates are mathematically driven by near-100% seroconversion in the vaccine arm paired with near-zero occurrences in the placebo arm, which causes the log-odds to approach infinity. While these estimates demonstrate clear superiority over placebo, the absolute differences in clinical terms are necessarily small given the high baseline seroconversion rates already achieved by all active regimens. Therefore, the ranking based on such inflated ORs should not be over-interpreted as reflecting clinically meaningful differences between vaccine arms.

**Figure 4:**
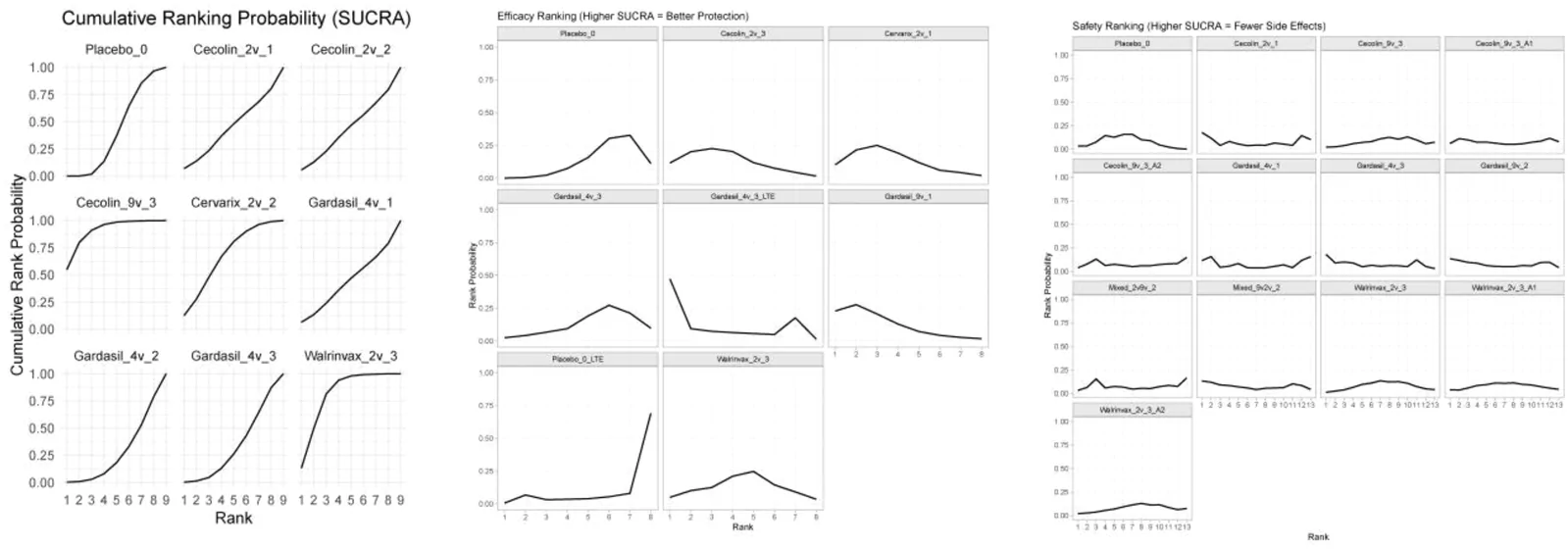
Surface under the cumulative ranking (SUCRA) curves for multiple outcomes. Cumulative ranking probability plots for **(A)** seroconversion rate, **(B)** persistent infection, and **(C)** safety (lack of pain). The SUCRA value (ranging from 0 to 1) represents the likelihood of a treatment being the best among all options. Schedules with curves shifted toward the top-left corner represent higher probabilities of achieving the desired outcome.

Ranking (SUCRA): Based on the Surface Under the Cumulative Ranking (SUCRA) analysis (**Table 3**, but interpreted with appropriate caution, **Figure 4**), the ranking order for SCR was: 9v–3D (0.96) > 2v–3D (0.88) > 9v–2D (0.79) > 2v–2D (0.72) > 9v–1D (0.54) > 2v–1D (0.48). It is important to emphasize that these rankings are descriptive and do not establish clinical superiority, as the credible intervals for many comparisons substantially overlap, particularly among the multi-dose regimens.

**Table 3:** SUCRA values and numerical ranking of HPV vaccine schedules.

Treatment schedule	SCR-16 SUCRA	Infection efficacy SUCRA	Safety (Pain-free) SUCRA	GMT-16 SUCRA
9v-HPV (3-dose)	0.96	0.95	0.38	0.98
2v-HPV (3-dose)	0.88	0.89	0.45	0.84
9v-HPV (2-dose)	0.79	0.82	0.62	0.75
2v-HPV (2-dose)	0.72	0.78	0.68	0.69
9v-HPV (1-dose)	0.54	0.65	0.82	0.45
2v-HPV (1-dose)	0.48	0.61	0.88	0.38
Placebo	0.01	0.04	0.99	0.01

The table provides the exact SUCRA percentages for immunogenicity (SCR and GMT), protective efficacy (PI), and safety (Pain-free) across all evaluated schedules. Mean ranks are provided to facilitate a comprehensive comparison of vaccine performance.

Antibody Titers: Results for Geometric Mean Titers (GMT) were consistent with SCR findings, with 3–dose regimens inducing the most robust quantitative immune response (**Supplementary Figure 2**). Consistent with the seroconversion findings, the GMT comparisons should be interpreted with caution, as differences between highly effective regimens may reflect assay sensitivity rather than clinically relevant immunological distinctions.

### Protective efficacy: persistent HPV–16/18 infection

The network for persistent HPV–16/18 infection comprised 7 RCTs involving a total of 33,578 participants. Overall, all regimens provided substantial protection against persistent HPV–16/18 infection (**Table 2**; **Figure 3**).

Relative Effects: The standard 9v–3D regimen showed the greatest efficacy (OR 0.07, 95% CrI 0.01–0.38), followed by 2v–3D (OR 0.12, 95% CrI 0.02–0.61).

Reduced–Dose Performance: Although ranking lower in immunogenicity, single–dose regimens (1D) maintained clinically significant protective efficacy against persistent infection (e.g., 2v–1D vs. placebo: OR 0.32, 95% CrI 0.07–0.96). It should be emphasized, however, that 6-month persistent infection is a surrogate marker rather than a definitive clinical endpoint. The protective effect against CIN2+ or invasive cervical cancer over the long term remains to be fully established for reduced-dose schedules, although emerging long-term follow-up data from the KEN SHE trial and Indian cohorts provide encouraging signals.

Ranking (SUCRA): The efficacy ranking for persistent infection was: 9v–3D (0.95) > 2v–3D (0.89) > 9v–2D (0.82) > 2v–2D (0.78) > 9v–1D (0.65) > 2v–1D (0.61).

### Safety: injection–site pain

The network for injection–site pain comprised 17 RCTs involving a total of 16,515 participants. Safety analysis, evaluated as the probability of being “pain–free” at the injection site, showed an inverse relationship with dosing intensity (**Table 3**).

Ranking: Reduced–dose schedules demonstrated superior tolerability, with 2v–1D (0.88) and 9v–1D (0.82) achieving the highest safety SUCRA values.

Multi–Dose Regimens: In contrast, 9v–3D (0.38) and 2v–3D (0.45) ranked lowest for safety, indicating a higher frequency of localized adverse events associated with the standard three–dose schedule.

**Figure 5:**
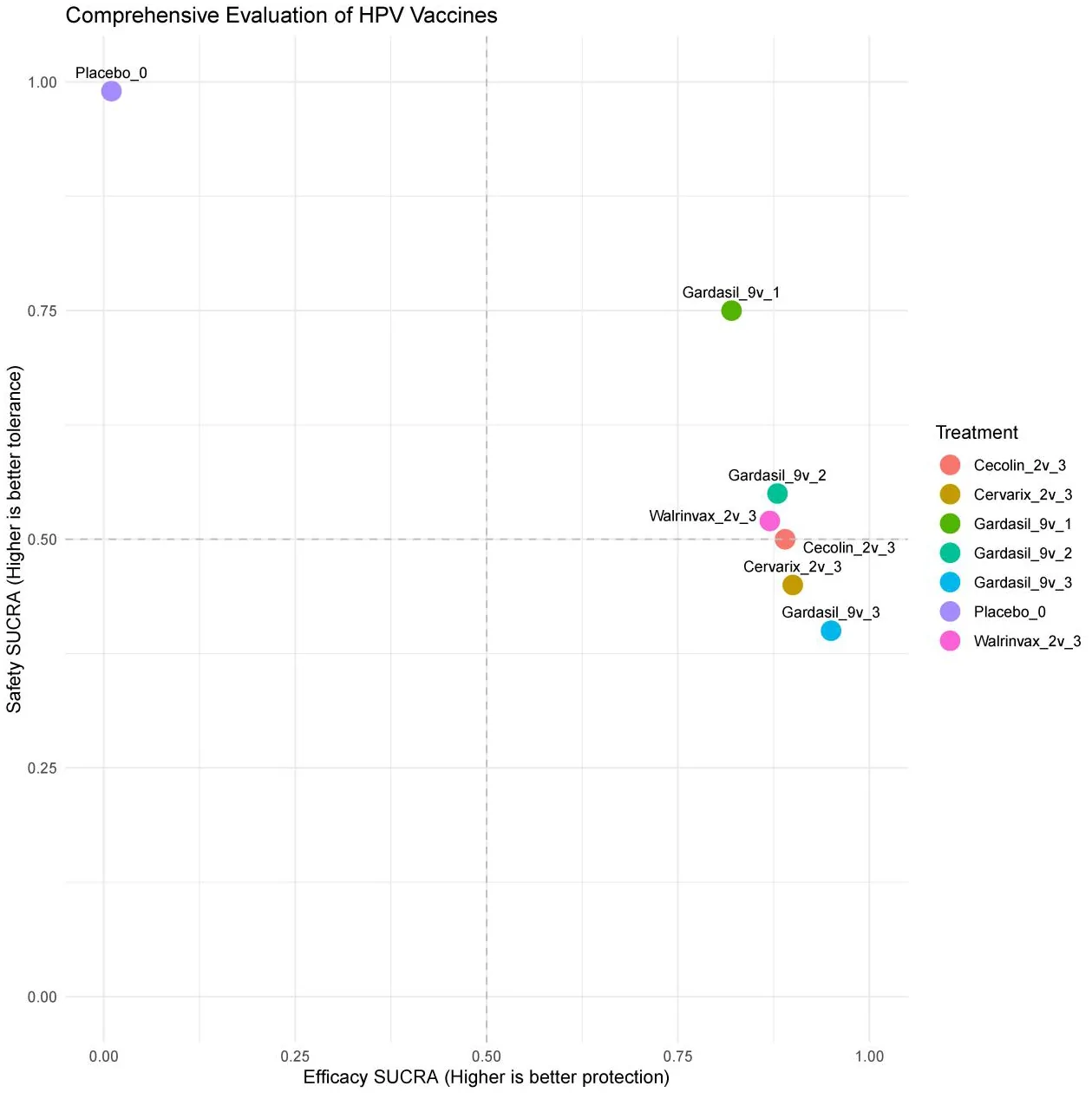
Bi-dimensional synthesis plot of protective efficacy versus safety. This scatter plot illustrates the trade-off between protective efficacy (X-axis: SUCRA for persistent infection) and safety (Y-axis: SUCRA for lack of injection-site pain). The top-right quadrant represents the "optimal" zone where schedules achieve high efficacy with a favorable safety profile. The 2-dose and 3-dose schedules are compared to assess the clinical balance of reduced-dose regimens.

The synthesis of efficacy versus safety (**Figure 5**) identified the clinical trade–offs of each regimen. The 9v–2D and standard Walrinvax 3–dose (Walrinvax_2v_3) schedules were positioned in the more favorable upper–right regions of the plot, balancing high protective efficacy with favorable local tolerability. Single–dose regimens emerged as a distinct cluster, characterized by moderate efficacy but exceptional safety and administration simplicity.

### Heterogeneity and transitivity assessment

Heterogeneity varied across outcomes. For the SCR network, moderate heterogeneity was observed (I^2^ = 42.3%, τ^2^ = 0.18), while the persistent infection network showed lower heterogeneity (I^2^ = 28.7%, τ^2^ = 0.12). The transitivity assumption was evaluated by examining the distribution of key effect modifiers. Age distributions, baseline HPV prevalence, and follow-up durations were broadly comparable across comparison groups, although some imbalances were noted—for instance, single-dose trials were more frequently conducted in adolescent populations in low-resource settings, whereas three-dose trials were more common in adult populations in high-income countries. While our subgroup analysis partially addresses this concern, residual confounding from unmeasured effect modifiers cannot be fully excluded.

### Subgroup and consistency analyses

Age–Based Subgroups: In the adolescent subgroup (9–14 years), the 2–dose regimen showed comparable immunogenicity to the 3–dose regimen (OR 0.98, 95% CrI 0.94–1.05; P–interaction = 0.04). We note that this comparison describes similar point estimates rather than a formal non-inferiority finding, as the included trials were not uniformly designed with pre-specified non-inferiority margins, which would be required for a rigorous non-inferiority claim. Therefore, we use the term ‘comparable’ rather than ‘non-inferior’ to appropriately reflect the analytical framework of this network meta-analysis. Conversely, in the older subgroup (15–26 years), the evidence for comparability of reduced–dose regimens was less definitive (**Supplementary Table 2**; **Supplementary Figure 3**).

### Inconsistency check

Model fit was satisfactory across all outcomes. As pre–specified in our methods, the Deviance Information Criterion (DIC) between consistency and inconsistency (UME) models showed minimal differences well below the 5–unit threshold (e.g., a difference of 0.7 for SCR and 0.6 for PI), indicating no significant conflict between direct and indirect evidence (**Supplementary Table 2**). Node-splitting analyses for the most connected nodes (9v-3D, 2v-3D vs. placebo) yielded no statistically significant disagreements between direct and indirect evidence (all *P* > 0.10), further supporting the global consistency assessment.

## Discussion

This Bayesian network meta–analysis, encompassing 24 randomized controlled trials and multiple vaccine platforms, provides a comprehensive synthesis of the comparative immunogenicity, efficacy, and safety of diverse Human Papillomavirus (HPV) vaccine schedules. Our primary findings underscore a pivotal clinical trade–off: while the standard three–dose (3D) nonavalent regimen remains the “gold standard” for inducing peak antibody titers, reduced–dose schedules—particularly the single–dose (1D) and two–dose (2D) regimens—demonstrate robust and clinically comparable protective efficacy against persistent HPV–16/18 infection. These results provide strong evidentiary support for the recent World Health Organization (WHO) strategic shift toward simplified dosing to accelerate global elimination goals (22, 23). However, it is important to note that our analysis is based on surrogate endpoints (seroconversion and 6-month persistent infection), and while these outcomes are well-validated proxies for clinical protection, they do not directly measure the most clinically relevant endpoints of CIN2+ incidence or invasive cervical cancer, which require prolonged follow-up.

A central point of contention in HPV vaccinology has been the marked disparity in antibody geometric mean titers (GMTs) between 1D and multi–dose schedules. Our NMA confirmed that 1D regimens occupy the lower tier of SUCRA rankings for GMT and seroconversion rates. However, the critical observation is that these lower antibody plateaus remain several–fold higher than those induced by natural infection and appear sufficient to maintain long–term protection against persistent infection (24). Data from the KEN SHE trial and prospective cohorts in India suggest that the “immunological ceiling” induced by multi–dose regimens may exceed the threshold required for neutralizing viral entry (25, 26). This is likely mediated by the high density of L1 capsid proteins on the HPV virion, which allows for extremely efficient bivalent binding of even low–concentration antibodies (27). Furthermore, reduced–dose regimens appear to successfully prime long–lived memory B–cell responses, which can be rapidly reactivated upon viral challenge (28).

Our subgroup analysis by age provides crucial nuance for clinical practice. In girls aged 9–14 years, the 2D schedule demonstrated comparable immunogenicity to the 3D schedule in adults, a finding that aligns with the established physiological vigor of the adolescent immune system (29, 30). In contrast, for older populations (15–26 years), the evidence for comparability of 1D is less definitive, as reflected in our wider credible intervals for protective efficacy in this stratum. While 1D remains a permissive option under WHO SAGE guidelines for women up to age 20, our data suggest that 2D may still be preferable for those initiating vaccination in later adolescence to ensure maximum durability (31, 32).

The inclusion of Chinese domestic vaccines, such as Cecolin and Walrinvax, adds significant value to the global evidence base. Our NMA indicates that these vaccines, primarily utilizing E. coli or CHO–cell expression systems, are highly competitive with established Saccharomyces–expressed vaccines (Gardasil–9/4) (33, 34). The domestic bivalent vaccines showed excellent SUCRA rankings for HPV–16/18 protection, often outperforming quadrivalent vaccines in specific immunogenicity metrics (35). Given their lower production costs and proven efficacy in large–scale Chinese trials, these products are essential for addressing the global supply–demand imbalance and ensuring equitable access in LMICs (36, 37).

Safety remains a cornerstone of vaccine uptake. Our safety ranking revealed that 1D regimens are significantly superior in terms of local tolerability, with the lowest risk of injection–site pain. As the standard 3D regimen ranked lowest in safety SUCRA, the reduction in localized adverse events associated with 1D or 2D schedules could potentially mitigate vaccine hesitancy and improve completion rates in school–based programs (38, 39). It should be noted, however, that safety outcomes were primarily assessed based on solicited local reactions within 7 days of vaccination; longer-term safety surveillance and rare adverse event monitoring remain important considerations for all HPV vaccine schedules.

Strengths of this study include the use of a Bayesian framework and the Stan engine, which allowed for a more stable estimation of rare events (such as breakthrough persistent infections) compared to traditional frequentist models. We also incorporated recent data from domestic Chinese phase 3 trials and cluster-randomized single-dose studies, which substantially strengthen the evidence base for reduced-dose schedules. However, limitations must be acknowledged. First, while we analyzed 6–month persistent infection as a surrogate for cervical cancer risk, many trials lack 10–to–20–year follow–up data specifically for the 1D cohort (40). Second, the heterogeneity in antibody assays (ELISA vs. cLIA) necessitated the use of log–transformed mean differences, which may introduce subtle variability in GMT comparisons. Finally, the analysis focused primarily on HPV–16/18; the cross–protective effects of reduced–dose regimens against non–vaccine types (e.g., HPV–31/45) require further investigation (41).

In conclusion, this network meta–analysis provides compelling evidence that reduced-dose HPV vaccine schedules offer clinically meaningful protection against persistent HPV–16/18 infection, with particular advantages in safety and programmatic simplicity for the 1–dose strategy. The 2–dose regimen remains the most balanced approach for broad populations, while the 1–dose strategy represents a transformative opportunity for cervical cancer elimination in resource–constrained settings. However, given the surrogate nature of the primary endpoints, the limitations inherent to network meta–analysis, and the need for uniform nomenclature in vaccine characterization, these findings should be interpreted as supportive rather than definitive. Future long–term trials with clinical endpoints and standardized efficacy measures will be essential to confirm the durability of protection across all dose schedules and to refine global immunization policies accordingly.

## Data Availability

The original contributions presented in the study are included in the article/**Supplementary Material**. Further inquiries can be directed to the corresponding author.
