# Supplementary material for: Immunogenicity, efficacy, and safety of different dose schedules of human papillomavirus vaccines: a Bayesian network meta–analysis of randomized controlled trials
Source: Front Immunol. 2026 Aug 6;17:1883217. doi: 10.3389/fimmu.2026.1883217 (PMC13490129; doi:10.3389/fimmu.2026.1883217)
Supplement: Supplementary file 1 [file DataSheet1.docx]

Supplementary Figure S1. Individual Risk of Bias Assessment (RoB 2.0) for the 24 Included Studies.

The "traffic light" plot details the risk of bias for each trial across five domains: randomization process, deviations from intended interventions, missing outcome data, measurement of the outcome, and selection of the reported result.
