# Supplementary material for: Immunogenicity, efficacy, and safety of different dose schedules of human papillomavirus vaccines: a Bayesian network meta–analysis of randomized controlled trials
Source: Front Immunol. 2026 Aug 6;17:1883217. doi: 10.3389/fimmu.2026.1883217 (PMC13490129; doi:10.3389/fimmu.2026.1883217)
Supplement: Supplementary file 2 [file DataSheet2.docx]

Supplementary Figure S2. Network Meta-Analysis of HPV-16 Antibody Titers (GMT).

(A) Evidence network for antibody Geometric Mean Titers (GMT). (B) Forest plot of Log Mean Differences (MD) compared with Placebo. (C) SUCRA ranking for antibody levels. GMT data provide a quantitative measure of immune response intensity.
