# Supplementary material for: Immunogenicity, efficacy, and safety of different dose schedules of human papillomavirus vaccines: a Bayesian network meta–analysis of randomized controlled trials
Source: Front Immunol. 2026 Aug 6;17:1883217. doi: 10.3389/fimmu.2026.1883217 (PMC13490129; doi:10.3389/fimmu.2026.1883217)
Supplement: Supplementary file 3 [file DataSheet3.docx]

Supplementary Figure S3. Subgroup Analysis of Immunogenicity and Efficacy by Age Group.

Comparative forest plots for young adolescents (aged 9–14 years) versus adults (aged 15–26+ years). This analysis evaluates whether the non-inferiority of 2-dose regimens is maintained across different age strata.
