# Supplementary material for: Immunogenicity, efficacy, and safety of different dose schedules of human papillomavirus vaccines: a Bayesian network meta–analysis of randomized controlled trials
Source: Front Immunol. 2026 Aug 6;17:1883217. doi: 10.3389/fimmu.2026.1883217 (PMC13490129; doi:10.3389/fimmu.2026.1883217)
Supplement: Supplementary file 4 [file Table1.docx]

| Supplementary Table S1. Detailed Search Strategy for All Databases | |  |
| --- | --- | --- |
| Database | Search Strategy (Keywords and MeSH Terms) | |
| PubMed / MEDLINE | #1. MeSH descriptor: [Human papillomavirus vaccines] explode all trees<br>#2. ("HPV vaccine*" OR "Human Papillomavirus vaccine*" OR "Gardasil" OR "Gardasil 9" OR "Cervarix" OR "Cecolin" OR "Walrinvax" OR "Cervavac")<br>#3. #1 OR #2<br>#4. ("dose*" OR "dosage*" OR "schedule*" OR "single dose" OR "one dose" OR "two dose*" OR "three dose*" OR "reduced dose*" OR "dose-reduction" OR "immunization schedule*")<br>#5. #3 AND #4<br>#6. ("randomized controlled trial" [pt] OR "controlled clinical trial" [pt] OR "randomized" [tiab] OR "placebo" [tiab] OR "clinical trials as topic" [mesh:noexp] OR "randomly" [tiab] OR "trial" [ti])<br>#7. #5 AND #6 | |
| Embase (Ovid) | #1. 'human papillomavirus vaccine'/exp<br>#2. ('hpv vaccine':ti,ab OR 'gardasil':ti,ab OR 'cervarix':ti,ab OR 'cecolin':ti,ab OR 'walrinvax':ti,ab OR '9-valent hpv vaccine':ti,ab)<br>#3. #1 OR #2<br>#4. ('dosage':ti,ab OR 'dose':ti,ab OR 'schedule':ti,ab OR 'single dose':ti,ab OR '1-dose':ti,ab OR '2-dose':ti,ab OR 'reduced dose':ti,ab)<br>#5. #3 AND #4<br>#6. ('randomized controlled trial'/exp OR 'randomization'/exp OR 'clinical trial'/exp OR 'double-blind procedure'/exp)<br>#7. #5 AND #6 | |
| Cochrane Library | #1. MeSH descriptor: [Human papillomavirus vaccines] explode all trees<br>#2. (hpv vaccine*):ti,ab,kw OR (gardasil):ti,ab,kw OR (cervarix):ti,ab,kw OR (cecolin):ti,ab,kw OR (walrinvax):ti,ab,kw<br>#3. #1 OR #2<br>#4. (dose* OR dosage* OR schedule* OR "single dose" OR "one dose" OR "two doses"):ti,ab,kw<br>#5. #3 AND #4 in Trials | |

Supplementary Table S1. Full Search Strategy and Terms for Electronic Databases.

Detailed search strings used for PubMed, Embase, and The Cochrane Library, including Medical Subject Headings (MeSH) and free-text keywords.
