# Supplementary material for: Immunogenicity, efficacy, and safety of different dose schedules of human papillomavirus vaccines: a Bayesian network meta–analysis of randomized controlled trials
Source: Front Immunol. 2026 Aug 6;17:1883217. doi: 10.3389/fimmu.2026.1883217 (PMC13490129; doi:10.3389/fimmu.2026.1883217)
Supplement: Supplementary file 5 [file Table2.docx]

| Comparison | Subgroup | Outcome (SCR) OR (95% CrI) | P-interaction |
| --- | --- | --- | --- |
| 2D vs 3D | Young (9-14y) | 0.98 [0.94, 1.05] | 0.04 |
| Adult (15-26y) | 0.92 [0.85, 0.98] |  |  |
| 1D vs 3D | Young (9-14y) | 0.92 [0.82, 1.02] | 0.01 |
| Adult (15-26y) | 0.72 [0.58, 0.85] |  |  |

Supplementary Table S2. Deviance Information Criterion (DIC) for Model Selection and Consistency Check.

Comparative statistics for fixed-effects versus random-effects models, and consistency versus inconsistency (UME) models. Smaller DIC values indicate superior model fit.
