# Supplementary material for: Immunogenicity, efficacy, and safety of different dose schedules of human papillomavirus vaccines: a Bayesian network meta–analysis of randomized controlled trials
Source: Front Immunol. 2026 Aug 6;17:1883217. doi: 10.3389/fimmu.2026.1883217 (PMC13490129; doi:10.3389/fimmu.2026.1883217)
Supplement: Supplementary file 6 [file Table3.docx]

| Outcome Metric | Consistency Model DIC | UME Model DIC | DIC Difference | Consistency |
| --- | --- | --- | --- | --- |
| SCR (HPV-16) | 62.4 | 63.1 | 0.7 | Satisfactory |
| Persistent Inf. | 45.2 | 45.8 | 0.6 | Satisfactory |
| Safety (Pain) | 58.7 | 59.5 | 0.8 | Satisfactory |

Supplementary Table S3. Sensitivity Analysis Excluding Studies with "Some Concerns" in Risk of Bias.

Results of the NMA after excluding trials with potential bias to ensure the robustness of the primary findings.
